# Characterization of students with high intellectual capacity: the approach in the Portuguese school context and importance of teacher training for their educational inclusion

**DOI:** 10.3389/fpsyg.2024.1196926

**Published:** 2024-03-01

**Authors:** Ramón García-Perales, Alberto Rocha, Ana Aguiar, Ana Isabel S. Almeida

**Affiliations:** ^1^Faculty of Education, Department of Pedagogy, University of Castilla-La Mancha, Albacete, Spain; ^2^Research Center in Psychopedagogy and Psychopedagogical Research [CIPsp], Buenos Aires, Argentina; ^3^ISCE DOURO- Instituto Superior de Ciências Educativas do Douro, Penafiel, Portugal; ^4^ESE IPVC-Instituto Politécnico de Viana do Castelo, Viana do Castelo, Portugal; ^5^Associação Nacional para o Estudo e Intervenção na Sobredotação (ANEIS), Braga, Portugal

**Keywords:** teacher training, inclusion, high intellectual capacities, school legislation, Portuguese educational system

## Abstract

This paper takes a terminological approach to the concept of high intellectual abilities, highlighting the distinctive aspects of the way it is addressed in the Portuguese educational context. It outlines the existing conceptual differentiation in the field of higher abilities, emphasizing how the main characteristics are described and including the strengths and weaknesses of current teaching and learning processes in Portugal. As we will show, educational work with these students in Portuguese schools is characterized by unequal regulation compared to other educational needs, by an imbalance of educational processes aimed at these gifted students, and by clear underdiagnosis that sometimes leads to them being ignored in the classroom, with the consequent harm that this can cause to their overall development and to their academic and professional careers. The paper emphasizes the importance of teacher training in Portugal as an aspect that could be key to reversing, as far as possible, this gap in educational processes—which currently include educational inclusion for all their students as part of the key action principles in the Portuguese educational system.

## Introduction

Education is a key element for strengthening a democratic, cohesive society. One of its many goals is to facilitate the inclusion of all students in the educational process with a view to anticipating and preventing the appearance of attitudes of exclusion and discrimination (González-Gil and Pastor-Martín, 2014; Vázquez et al., 2020; Torres, 2022). This is necessary regardless of each student’s personal characteristics (Alves, 2019; Espada et al., 2019) or whatever circumstances may be hindering their learning (Arnaiz, 2019; Reyes-Parra et al., 2020). Individualized attention will help lead to a positive working atmosphere in the school and therefore, a more inclusive, integrated society.

Traditionally, education for students with special needs was marked by ignorance and discrimination, and they were often relegated to the background (Arnaiz et al., 2020). Social and educational movements over recent years have helped to change that segregationist approach in Portugal. There has been significant progress in the field, and nowadays the Portuguese educational system prioritizes the principles of inclusion and equity in their educational policies, leading to progress in the democratization of education. This integration has led to the generalization of numerous measures and actions that are increasingly incorporated into educational contexts (Meijer and Watkins, 2019; Jia et al., 2022), including that of Portugal (Alves, 2019).

These measures and actions are put into practice by teachers in collaboration with other educational agents. Portuguese education professionals must be aware that a basic premise of their educational praxis is to ensure the inclusion of all their students (Colmenero et al., 2015; Polo and Aparicio, 2018; Alves, 2019; Miller et al., 2022; Torres, 2022), promoting increasingly integrative school practices regardless of students’ characteristics and potential. In this regard, teachers become mediators of inclusive, equitable, high-quality teaching and learning processes, and they must be aware that each person is unique, and everyone’s potential must be addressed individually in day-to-day education. In Portugal, teachers with more training and experience in inclusion show more positive attitudes toward the development of inclusive educational practices (Torres, 2022).

This teaching role in students’ inclusion has been underscored by various studies (Alves, 2019; González-Gil et al., 2019; Perlado et al., 2019; Pérez-Gutiérrez et al., 2021; Torres, 2022; Yada et al., 2022), including studies referring to education for highly intellectually able students (García-Perales and Almeida, 2019; Ezzani et al., 2021; García-Perales and Jiménez-Fernández, 2022; Piske et al., 2022; Rocha et al., 2022).

Teacher training is crucial to adequately supporting students with high intellectual abilities and promoting equity in education (Sayı, 2018), considered as key in initial teacher training in Portugal (Martins et al., 2020). Most of the time, Portuguese teachers are not given specific training to teach students with high intellectual abilities (Antunes et al., 2020), which can lead to under-identification and these students’ being undervalued. Furthermore, teachers without adequate training may not be able to provide the necessary challenges and learning opportunities for highly intellectually able students. Teacher education in Portugal can help fill this gap and enable teachers to properly identify and support students with high intellectual abilities (Fleith et al., 2010).

With proper training, teachers can learn to create distinctive learning environments, provide opportunities for enrichment and acceleration, and adapt the curriculum to meet these students’ needs. At the same time, teacher training can help to promote equity and social justice in Portuguese education (Torres, 2022), as students with high intellectual abilities often come from different socio-economic and ethnic backgrounds. By receiving proper training, teachers can be better prepared to deal with the diversity of these students and provide a fairer, more inclusive educational environment (Sayı, 2018).

This article examines educational inclusion of highly intellectually able students, mainly in the Portuguese educational context. In order to understand how education for these students is delivered in Portugal, teaching is considered as the core work to analyze, in order to address factors from teaching practice that might affect the educational response directed toward these gifted children. Understanding these factors could be key in bridging the current gap in Portuguese education aimed at these students. In line with this, in the final part of the article we underscore the importance of teacher training as a key element in combatting the current discrimination in how these students are educated.

## School inclusion in the Portuguese educational system

The legislation establishing the legal framework for Inclusive Education was approved in 2018, through Decree-Law No. 54/2018 (Ministério da Educação da Portugal, 2018). It calls for inclusive schools, which include all students, with access to education and training for everyone, moving away from the narrow concept of Special Educational Needs and rejecting the idea that intervention needs categorization. On the contrary, the focus is on the education the school delivers to all students (Saragoça and Candeias, 2019), with guidelines based on universal design and a multilevel approach (Antunes et al., 2020).

The importance of this Decree in promoting inclusive education is undeniable, anchored in Sustainable Development Goal number 4, which aims to ensure access to inclusive, quality, equitable education and promote lifelong learning opportunities for all (Naciones Unidas, 2015). It also reiterates the concept of inclusion, which runs through the Salamanca Statement (Naciones Unidas, 1994)—a statement that expounds the holistic, balanced view that includes “disabled children and gifted children, children living in the street and working children, children from remote or nomadic populations, children from linguistic, ethnic or cultural minorities and children from other disadvantaged or marginalized groups or areas” (p. 6).

It is in this context that we propose a revision of Decree-Law No. 54/2018 (Ministério da Educação da Portugal, 2018), which makes no reference to measures for highly able students or their characteristics. Despite this omission, article 28 of Regulatory Circular no. 1 – F/2016 (Ministério da Educação da Portugal, 2016), regulating evaluation and certification of learning for students, and the measures that can be adopted for follow-up and learning delivery, focuses on particular cases where students who demonstrate exceptional learning ability and suitable levels of maturity can be moved up.

In our view, another reading of Decree-Law No. 54/2018 (Ministério da Educação da Portugal, 2018) is possible, one which is broader and more positive, although clearly a document of this nature will have the life and interpretation that schools generally, and teachers in particular, want to give it. In this regard, it is essential in this Portuguese educational context to reduce the existing gap between educational legislation and inclusive teaching practice, so that teacher training in educational inclusion is an essential means of implementing inclusive pedagogical strategies (Torres, 2022).

On the one hand, and sharing the view of Miranda and Almeida (2019) that an inclusive education cannot be restricted to deficit, one potential reading of the Decree is that it never uses the word “gifted.” Indeed, this omission shows both the scant public policy investment in terms of giftedness and the lack of commitment in terms of the legal framework, which goes against the Decree itself—which values and supports inclusive schooling, equal opportunities, and pedagogical differentiation.

In addition, it seems pertinent to remember that the same document defines inclusion as a political priority embodying the “right of each student to an inclusive education that responds to their potential, expectations and needs within the scope of a common and plural educational project that provides everyone with participation and a sense of belonging in effective conditions of equity, thus contributing decisively to higher levels of social cohesion” (p. 2918). The decree illustrates a broad understanding of an inclusive school as one that is capable of including everyone despite their differences, whether in talent or potential, or difficulty participating in the standard curriculum; differentiated paths, that “allow everyone to progress in the curriculum with a view to their educational success” (p. 2919), ensuring every student finishes compulsory schooling (Ministério da Educação da Portugal, 2018). Dealing with gifted students as well as students with other specific needs means inclusive educational practices.

So despite giftedness not being mentioned anywhere in the Decree, what we would like to reiterate, contrary to what might be expected after reading the first few lines of the document, is that from our point of view, the constant references to the potential of diversity suggest inclusion of talents and giftedness. Evidence for that conviction lies in article 1 of Decree-Law No. 54/2018 (Ministério da Educação da Portugal, 2018), which defines the purpose and scope of Inclusive Education, stating that “this Decree-Law establishes the principles and standards that guarantee inclusion as a process which aims to respond to the diversity of needs and potential of each and every one of the students, through greater participation in the learning process and in the life of the educational community” (p. 2919).

Other evidence includes the theoretical assumptions the Decree stresses, namely, the insistence on adapting the teaching process to the individual characteristics of each student. This means deploying various means which in turn requires commitment to the autonomy of schools and education professionals—who must be trained to develop skills that accompany curricular diversification, “taking each and every one to the limit of their potential” (Ministério da Educação da Portugal, 2018, p. 2919). Similarly, equity and personalization are two of the guiding principles of inclusive education (*cf.* article 3 of Decree-Law no. 54/2018, Ministério da Educação da Portugal, 2018), which reiterates the need to individualize educational processes, an essential principle in educating gifted students (Rocha et al., 2021).

Turning to the realization of these theoretical assumptions, and in terms of methodological options, there is once again no reference to measures in the context of giftedness. However, there are measures to support learning and inclusion based on a universal design and a multilevel approach, which aim to adapt to each student’s needs and potential. We return to the words of Rotigel and Fello (2004), who affirmed that talented or gifted students in a certain area require teaching methodologies that promote learning with greater depth of content and more complex problems to tackle instead of tasks that require learning by repetition and practice.

Some more legal support is necessary, similar to the education policy in the Autonomous Region of Madeira, which includes legislation for gifted and highly able students. This is exemplified by Regional Legislative Decree No. 33/2009/M, of December 31, which establishes the legal regime for special education, the transition to adult life and rehabilitation of people with disabilities in the Autonomous Region of Madeira, and particularly article 1, point 2, indicating the importance of measures in the field of early care and high intellectual capacities (Ministério da Educação da Portugal, 2009).

In fact, there are studies that have highlighted the importance of educational policies and practices that recognize and support these highly able students in Portugal, not only to promote their academic and personal success, but also to improve equity and social justice in the Portuguese educational system (Miranda and Almeida, 2018; Antunes et al., 2020). Therefore, it is essential that Portuguese education policy explicitly recognizes the importance of supporting these students and provides clear guidance on how schools can do so.

In addition, the inclusion of highly able students in the Portuguese educational system is a challenge that requires suitable attention and training on the part of teachers. Some authors have highlighted the need for Portuguese teachers to receive specialized training about these students’ needs so that they can properly identify and support them in the classroom (Miranda and Almeida, 2018; Martins et al., 2020; Reis-Jorge et al., 2021). Therefore, Portuguese educational policy needs to provide clear guidelines and resources for teacher training in relation to gifted students’ needs.

## Giftedness: conceptualization and distinctive characteristics

Various authors have focused on identifying a specific definition of the term giftedness. The literature ranges from the search to explain what triggers the phenomenon and possible ways to analyze it, to identification of typical characteristics, different profiles, and respective manifestations. This is increasingly necessary research given the importance of intervening with children, young people, and adults with these skills who deviate from typical development.

Giftedness, in general, includes intellectual components, as well as other variables associated with psychological (for example, personality) and social (for example, value system) factors (Rocha et al., 2020). The importance of each variable in the development and expression of high abilities varies according to the different ways of analyzing this potential, from theories that address more cognitive components (for example, Renzulli’s Three Rings Theory), to others which cover socio-emotional and contextual components (for example, Gagné’s differentiated model of giftedness and talent), and cultural components (for example, Pfeiffer’s tripartite model).

Based on the characteristics, there are four main profiles for categorizing this potential. They are intellectual precocity, giftedness, talent and double exceptionality. Giftedness seems to be identified with high capacities or talents, with the intellectual and academic areas also including the artistic area, sports, scientific research and business management. These capacities emerge in favorable contexts of learning and development and are expressed in combination with other psychological constructs such as personality, creativity, motivation or value systems.

The first profile refers to children who exhibit above-average ability at a preschool age and early development of psychomotor, cognitive (for example, language or numerical reasoning), metacognitive, linguistic, and/or socio-emotional abilities (Engle et al., 2007; Guenther, 2011; Martins, 2013; Bildiren, 2017). These children usually have a large vocabulary and demonstrate creativity in their play, seeking to acquire new knowledge and skills, along with exhibiting constant curiosity and a need to understand everything around them. They ask more complex questions about more advanced content that their parents or peers do not usually show an interest in Kwiatkowska (2020). Some children may even acquire skills such as reading, writing, or more complex mental arithmetic early, while others may excel in other areas where early skill acquisition is not as noticeable (Bildiren, 2017).

Giftedness is identified as above-average potential from school age onwards, with development of skills and acquisition of knowledge that produces exceptional performance in various domains of activity (Renzulli, 2018; Gagné, 2018a,b). Many studies have attempted to provide good explanations of this phenomenon, and initially there was a complete association with intellectual abilities, that is, a focus on higher intelligence. However, in recent years research has shown that giftedness is not only associated with IQ, but also covers other areas of human potential such as creativity, socio-emotional, sensory, and motor skills (Gagné, 2018a), and that people can excel in non-academic areas such as artistic performance and sport, or in their interpersonal skills, among others (Rocha et al., 2020).

This raises the need to reinforce other variables in addition to IQ. Renzulli (1978, 1986) suggested an analysis associated with the interaction of three components, namely, above-average ability (from general to specific), creativity (potential for the creation of products, originality of ideas and innovation of actions) and motivation or commitment to the task (great focus, interest and persistence in the acquisition of knowledge and/or achievement orientation). This way of analyzing giftedness makes it easier to identify people with both intellectual and creative potential. More recently, Renzulli (2018) reinforced the idea that there are two main profiles of giftedness, academic and creative/productive. The former refers to the manifestation of superior intellectual abilities that stand out in the academic context, with performance assessed in intelligence and aptitude tests. In the latter, above-average abilities are applied in other areas of greater personal interest, in the creation of original and innovative products, not necessarily connected to the school curriculum (Renzulli, 2018).

Talent is associated with capabilities that develop over time and are subject to the process of learning and constant practice (Gagné, 2004, 2007). Gagné’s (2009) Differentiated Model of Giftedness and Talent (MDST) arose from the need to distinguish talent from giftedness.

This type of high capacity is distinguished from giftedness, since it refers to a field of human activity based on transforming aptitudes into systematically trained competencies (knowledge and skills), which can be extremely varied (for example, sports and music), as opposed to giftedness which is characterized by natural, untrained, spontaneously expressed aptitudes in one or more domains; either intellectual, creative, socio-affective or sensorimotor (Gagné, 2004, 2018a). Following this idea that each person can manifest their potential in one or more areas, it is worth highlighting Gardner’s (1999) Multiple Intelligence Model, which describes seven main domains of intelligence, linguistic, logical-mathematical, kinesthetic, interpersonal, intrapersonal, spatial, musical, and naturalistic, in addition to other additional dimensions such as spiritual, moral, and existential intelligence. More recently, Gagné recognized that family and school are two crucial contexts for optimum development of cognitive abilities (Gagné, 2018b).

In addition, it is worth noting Pfeiffer’s (2013) work emphasizing the importance of including cultural components in the analysis of high abilities. According to Pfeiffer’s (2013, 2019) Tripartite Model, potential is perceptible when people manifest notable results or productions in one or more areas valued in their culture and society, based on their experiences and opportunities.

Finally, the double exceptionality profile is a difficult concept to explain, since it is associated with a continuum with two poles, with high capacities at one end and one or more neurodevelopmental disorders at the other (Baum et al., 2017). Thus, double exceptionality refers to people who show achievement potential in one or more domains and who, simultaneously, show one or more neurodevelopmental disorders (for example, Specific Learning Disorder, Autism Spectrum Disorder, Hyperactivity and Attention Disorder) (Baum et al., 2017; Gierczyk and Hornby, 2021).

Despite categorizing high abilities into these profiles, and although there is a huge variety in the way they are expressed, there is a set of cognitive and socio-emotional characteristics that are usually associated with high abilities, as Table 1 summarizes.

**Table 1:** Characteristics associated with high intellectual capacities.

Cognitive*	Socioemotional*
Demonstrates unusual curiosity. Eager for new knowledge. Exhibits great logical reasoning. Learns quickly and easily. Has an excellent memory. Is observant and attentive to details. Likes to question and think critically. Demonstrates insight. Is able to generate many ideas. Uses unusual vocabulary for their age. Is imaginative or extravagant. Interested in very diverse topics. Likes to innovate and find new solutions to problems. Is fascinated by complex subjects. Demonstrates ease in acquiring and applying knowledge. Finishes tasks quickly. Has very specific and/or unusual interests for their age. Presents unusual ideas and/or gives original answers to problems.	Focuses a lot on tasks that interest them. Likes to be challenged to go further. Is empathetic and sensitive to what others feel. Demonstrates great persistence and/or stubbornness. Has unusual moral and ethical concerns for their age. Uninterested in routine or repetitive tasks. Seeks knowledge autonomously. Interested in social and cultural issues. Likes to work freely and independently. Seems quite mature for their age. Enjoys interacting with older children and/or adults. Demonstrates high vocational aspirations.

* From Associação Nacional para o Estudo e a Intervenção na Sobredotação (2021).

All this potential associated with high intellectual capacities needs favorable learning and development contexts in order to be perceived (Rocha et al., 2020). However, there is a huge gap in the socio-educational responses to these people, especially due to school settings with limitations ranging from a lack of professional knowledge—particularly in teachers (Ivarsson, 2023)—to the resources needed to meet these students’ needs.

## Strengths and weaknesses in the educational response

**Figure 1:**
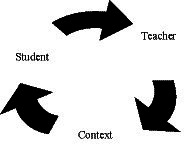
Dimensions of analysis.

Teaching is a complex task with a multitude of elements and circumstances that can have an impact on it. Teachers’ work nowadays is multidimensional and interdisciplinary, with increasingly heterogeneous educational processes involving greater participation of professionals from various fields (Alves, 2019; Torres, 2022). Bearing this in mind, elements can be defined within teaching practice that may promote or hinder the educational response aimed at highly intellectually able children in Portugal. We emphasize the role of the teacher in the individualization of teaching and learning processes for all of their students in order to ensure a personalized academic trajectory. We will define three areas of study to analyze how that education is delivered: students, teachers, and context—in terms of the role of the educational administration, social background, and family (Figure 1).

In the following section, we outline the strengths and weaknesses of how highly able students are educated in order to clarify these children’s reality, considering the teaching perspective. All the aspects indicated could be taken into consideration for generalization of educational policies by the responsible authorities to plug the current gap in terms of the educational response for these children.

## Strengths

The first of the strengths, related to students, is that their education is a fundamental right that must allow comprehensive development (Naciones Unidas, 1989, 2015; Torrente et al., 2022). This means that there must be a universal, compulsory, free period of schooling for everyone. It also means that every teacher has to tailor educational processes to the characteristics and potential of all the students in their classrooms (Alves, 2019). They must be aware of their students’ existing needs to promote full, comprehensive development. That includes those with high intellectual capacities, who must be considered in individualization of teaching and learning (Antunes et al., 2020).

In terms of the second area, the teacher, it is worth noting their general predisposition toward inclusion and individualized education for all their students. They generally have favorable attitudes toward inclusion (Álvarez-Castillo and Buenestado-Fernández, 2015; Sharma and Jacobs, 2016; Torres, 2022). Despite this, there is still a long way to go (Nilsen et al., 2019; Sundqvist and Hannås, 2021), particularly in the case of highly intellectually able children (Martins et al., 2020), as we will see below. In addition, teachers demand more training, and training that is varied and suited to current conditions so that they can deal with the full range of characteristics in their classrooms (Collado-Sanchís et al., 2020; Florian and Camedda, 2020; Quesada, 2021; Falla et al., 2022). Teachers’ roles as dynamic elements of social transformation is undeniable (Nilsen, 2017), which makes it essential to examine how teacher empowerment can be improved to cope with the inherent diversity in educational processes.

Finally, in relation to contextual aspects, it is important to highlight the necessary opening of schools to families and external services. On the one hand, the family is the key area for children’s primary socialization, so establishing good communication channels with teachers is essential. This makes the schools’ role of offering advice and guidance to families fundamental, including families of highly intellectually able children, who need specific guidance that teachers must know and must know how to communicate. External services, such as support groups and associations, also have an undeniable influence on educational processes, meaning that teachers must be able to develop relationships with them and find suitable points for dialogue, coordination, and joint reflection. At present, there are associations and groups serving children with high intellectual abilities and their families, and teachers should be aware of any treatment they receive outside of school so that any interventions are complementary and mutually inclusive. In this necessary collaboration between families and external services, it is worth highlighting the role of ANEIS in Portugal, an association with an essential role related to the educational, social and family response aimed at these students with higher abilities.

## Weaknesses

In contrast to the aspects that might help these students’ education in Portugal, weaknesses are elements that might make their schooling more difficult. We use the same starting points of student, teacher, and context.

In relation to the school, it is worth highlighting aspects related to how high intellectual ability is conceptualized, the prejudices and stereotypes there are about highly intellectually able people, and discrepancies in the numbers of identified cases. When it comes to conceptualization, operational terminological clarification is essential (Tourón, 2020), allowing differentiation between potential and talent in order to understand the heterogeneity in this group of students (Mendioroz et al., 2019). Prejudices and stereotypes must be considered (Reis-Jorge et al., 2021), bearing in mind that these highly able children exhibit a different understanding of the world that can make them feel or live differently to others. Teachers must be aware that these children often demonstrate performance that is not in line with their potential, they may not show interest in, or be motivated by, all the activities presented to them, they can make mistakes in simple tasks, exhibit poor study habits, and present an emotional maturity that does not fit with their age. Teachers should analyze these conceptions as a basic aspect in improving their professional development (Mendioroz et al., 2019; Reis-Jorge et al., 2021; Klimecká, 2022). In addition, these prejudices and stereotypes may lead, not only to difficulties in identifying highly able students and educational intervention, but may also contribute to the appearance of disorders such as personality disorders (Baudson and Preckel, 2016). Finally, the current discrepancy in the prevalence of cases appears to be characterized by underdiagnosis, which is even more striking for girls (Antunes, 2019; García-Perales and Jiménez-Fernández, 2022), with the numbers of cases identified still insufficient (Antunes, 2019; García-Perales and Almeida, 2019; García-Barrera et al., 2021). It is essential to generalize the processes for identifying these students in an educational system that seeks inclusion for all its students (Pfeiffer, 2017; Flynn and Shelton, 2022), this reality is also evident in the Portuguese educational system (Antunes et al., 2020; Martins et al., 2020).

Turning to teachers, it is important to note the weakness of their training for dealing with everything involved in these children’s education, diagnosis and intervention, and the inconsistency of educational processes aimed at them (Almeida et al., 2016). Both initial training of teachers and their continuing professional development are essential to adapt and optimize teaching to the educational, social and cultural demands of each point in time. Teacher training in terms of high intellectual abilities has occasionally been scarce and deficient (Gali et al., 2017; Martins et al., 2020; Barrera-Algarín et al., 2021; García-Barrera et al., 2021), which has led to educational processes that are a poor fit to these students’ potential (Reis and Renzulli, 2010). Teacher training affects the perceptions and expectations in relation to these students’ education (Conejeros-Solar et al., 2013; Reis-Jorge et al., 2021). Generalizing quality training processes on this subject is essential (Muglia and Tonete, 2016), in both initial training and continued development (García-Barrera et al., 2021). The poor training and the preconceptions mean that teachers demonstrate an inconsistent approach to how these students are educated (García-Perales and Jiménez-Fernández, 2022). That inconsistency is generally characterized by poor planning and being a poor fit to these children’s characteristics and potential, along with a lack of protocols for teachers to follow. This is all the more striking when considering that these children’s education should be an essential part of educational processes (Böker, 2021), and that these are the students who will be called on to lead change and drive innovation.

Lastly, in terms of contextual weaknesses, it is important to note the unequal regulation, the guidance and advice given to parents, and the involvement of external services in schools. The legislative frameworks covering education for these students vary widely between regions and between countries, or simply do not exist (García-Perales and Jiménez-Fernández, 2022), as specifically pointed out with Decree-Law No. 54/2018 in Portugal. This leads to inefficient education that is a poor fit to the students’ potential. When it comes to advice and guidance for families, teachers not having the proper training and skills might contribute to the spread of prejudices and stereotypes noted previously (Reis-Jorge et al., 2021). Families are key in the teaching and learning processes in the Portuguese school environment. Establishing suitable channels of communication and collaboration between the school and the family will affect students’ wellbeing, and that includes highly intellectually able students (Rocha et al., 2020; Peebles et al., 2023). Finally, Portuguese teachers sometimes view the involvement of external services in schools with suspicion or as a threat. These expectations should be changed into attitudes that are more open to dialogue and to working together. Collaboration between those in the Portuguese educational community is fundamental, regardless of whether they are internal or external to the school or whether they are strictly educational or not (Valentim et al., 2023).

## The importance of teacher training

Alongside the weaknesses of the educational response to the needs of highly intellectually able students and the emergence of more comprehensive legislation that recognizes the peculiarities of this group (Torrano and Sánchez, 2014; Tourón and Freeman, 2017), the central role of the teacher has been highlighted (Guenther, 2012; Belur and Oguz-Duran, 2017), an aspect that has been examined in the Portuguese educational context (Miranda and Almeida, 2018; Martins et al., 2020; Reis-Jorge et al., 2021).

In fact, working with this specific group requires educational practices that promote development of their abilities, recognizing that some of these students present difficulties (Tourón and Pfeiffer, 2015) that can lead to poor adjustment at school, rejection, or exclusion when their specific educational needs are not met. Students who have high intellectual abilities can demonstrate cognitive or non-cognitive characteristics (Zaia et al., 2018), and both must be given the same level of importance in diagnostic and intervention processes. There are many obstacles to overcome in terms of inclusive educational practices (Arnaiz, 2019; Spandagou, 2021), especially the need for training (Martins et al., 2020; Scanlon et al., 2022). As Matos (2015) showed in a Portuguese study about teacher training in relation to highly able or gifted students, teachers do not feel sufficiently trained to work with these students, and even exhibit a certain lack of interest in educating them.

Based on these assumptions, we accept that it is each teacher’s responsibility to change the educational practices prevailing in the classroom (Antunes et al., 2020), promoting inclusive measures and “pedagogical leadership favorable to excellence” (Antunes et al., 2020, p. 131). At the same time, there is an urgent need to create proper educational responses for all students (Syed and Ozbilgin, 2015), adapting and personalizing educational practices (Zeichner, 2018). This accountability is, in our view, anchored in attitudes that value differentiated and differentiating pedagogical dimensions, attitudes that recognize the need for instruments which promote pedagogical differentiation and serve highly able and gifted students (Antunes et al., 2020).

However, this inclusive teaching, based on a flexible approach to the curriculum, means reorganizing the educational space (López, 2020) at the macro and micro level, which in turn presupposes appropriate teacher training (Martins et al., 2020).

In fact, the many needs and challenges nowadays mean a need to invest in training for teachers (Pacheco, 2019), who, particularly when it comes to highly able students, know little about the subject and ignore intervention (Mendonça et al., 2020).

This complexity of this educational reality was underlined by González-Rivera (2022), who emphasized permanent training for education professionals. International organizations have also emphasized this, such as UNESCO, which highlights the importance of teacher training to meet the Sustainable Development Goals in the 2030 Agenda (Naciones Unidas, 2015). This underscores the need for teacher training with a humanistic approach and learning potential, which allows teachers to fully develop their students’ potentials (González-Rivera, 2022).

It is from this perspective that we support the training of specialist teachers, especially when it comes to dimensions related to identification and intervention for highly intellectually able students so that each teacher can: (i) understand these students’ characteristics; (ii) respond to their learning needs; (iii) be familiar with instruments used to identify these students; (iv) meet associations/experts that support gifted students; (v) understand and implement educational measures/pedagogical strategies for these students.

Teachers at every educational stage must be aware of this reality, which necessarily means including these topics in the initial teacher training curriculum, but also investing in training and planning ongoing training (García-Perales and Jiménez-Fernández, 2022). As the study in Portuguese schools by Lança (2014) demonstrated, much of the didactic knowledge on giftedness came from scientific events. Therefore, there is a clear need to generalize the processes of initial and continuous teacher training related to high intellectual abilities in Portugal (Matos, 2015; Antunes et al., 2020).

## Conclusion

The multidimensionality of high abilities means it is possible to identify a set of distinctive characteristics, some of which are more inherent to superior ability itself and others which are more associated with development and learning opportunities, not always for the best. The development of high intellectual abilities needs favorable individual and environmental conditions, which are not always present. It does not depend exclusively on the personal variables of the individual (Belur and Oguz-Duran, 2017; Mendonça et al., 2020; Lo et al., 2022; Peebles et al., 2023; Valentim et al., 2023), but instead it is clear that school, family, society, and culture play an essential role in this process, and can either help or hinder it.

Teachers are essential to this adjustment (Conejeros-Solar et al., 2013; Falla et al., 2022; Sigstad et al., 2022). Every educational measure needs trained teachers to implement it, and we must be aware that Portuguese teachers sometimes feel frustrated by the existing limitations to achieving quality, inclusive education, and they call for greater recognition and greater collective investment in inclusion, since this objective cannot fall only on their shoulders (Torres, 2022).

Highly intellectually able students tend to learn more, and learn fast, which requires deep, diversified, accelerated teaching, and a challenging, more cognitively-complex curriculum. In general, all educational measures call for teachers to master the content they teach and have the teaching skills to be able to promote inductive learning methods and help their students develop self-efficacy and intrinsic motivation (Martins et al., 2020). Knowledge and perceptions about these students’ education are essential aspects (Ivarsson, 2023).

In line with this, it is important to pay serious attention to individual abilities, interests and learning styles, with a focus on self-directed and independent learning, and less attention to characteristics such as age or school year. This means that teacher training is important and necessary in this area (Mendioroz et al., 2019; García-Barrera et al., 2021), something that has also been observed in the Portuguese educational context (Fleith et al., 2010; Martins et al., 2020). There needs to be raised awareness and training for all involved in education in order to create positive environments for these students and put measures into place that stimulate them and encourage their overall development (Baudson and Preckel, 2016; Rocha et al., 2020; Barrera-Algarín et al., 2021; Torrente et al., 2022; Peebles et al., 2023).

In a society that is more tolerant of differences, it is important that schools and teachers know how to respect those differences through different teaching and assessment practices (Arnaiz et al., 2020; Torres, 2022). When highly intellectually able students experience school failure, despite their abilities, we infer that the school has become dysfunctional and unattractive for these students. No principle of justice is fulfilled by pretending to treat all students equally when, in reality, they are all different!

An inclusive school must know how to equip itself with the technical means and the human resources needed for effective differentiation of its educational practices, as this is the only way to respect individual differences and serve everyone (Martins et al., 2020; Klimecká, 2023). The fundamental goal is that highly intellectually able students find, in their families, their schools, and their community, stimulus for fulfillment and personal excellence.

## Data availability statement

The original contributions presented in the study are included in the article/supplementary material, further inquiries can be directed to the corresponding author.

## Author contributions

RG-P, AR, AA, and AISA have participated in writing, revising and submitting of the article to the journal. All authors contributed to the article and approved the submitted version.
